# The Enhanced Interhemispheric Functional Connectivity in the Striatum Is Related to the Cognitive Impairment in Individuals With White Matter Hyperintensities

**DOI:** 10.3389/fnins.2022.899473

**Published:** 2022-06-28

**Authors:** Huahong Zhu, Ruomeng Qin, Yue Cheng, Lili Huang, Pengfei Shao, Hengheng Xu, Yun Xu, Qing Ye

**Affiliations:** ^1^Department of Neurology, Nanjing Drum Tower Hospital, Clinical College of Nanjing Medical University, Nanjing, China; ^2^State Key Laboratory of Pharmaceutical Biotechnology, Department of Neurology, Nanjing Drum Tower Hospital, Nanjing University Medical School, Nanjing, China; ^3^Institute for Brain Sciences, Nanjing University, Nanjing, China; ^4^Jiangsu Key Laboratory for Molecular Medicine, Nanjing University Medical School, Nanjing, China; ^5^Jiangsu Province Stroke Center for Diagnosis and Therapy, Nanjing, China; ^6^Nanjing Neurology Clinic Medical Center, Nanjing, China

**Keywords:** white matter hyperintensities, voxel-mirrored homotopic connectivity, cognitive impairment, cognitive heterogeneity, functional magnetic resonance imaging

## Abstract

**Objective:**

The cognitive performance of individuals with white matter hyperintensities (WMH) tends to vary considerably. This study aimed to explore the relationship of the synchronous spontaneous activities in homotopic areas across hemispheres, named as voxel-mirrored homotopic connectivity (VMHC), with the cognitive performance of individuals with WMH.

**Materials and Methods:**

Eighty-two WMH subjects without cognitive impairment (CI), 56 WMH subjects with CI, and 92 healthy subjects (HS) underwent neuropsychological tests and multimodal magnetic resonance imaging scans. VMHC maps were analyzed among the three groups. Correlative analyses were performed between VMHC values and cognitive function.

**Results:**

No significant difference in WMH volume, brain volume, or gray matter atrophy rate was shown between WMH subjects with and without CI. In contrast, those with CI displayed lower VMHC in the bilateral cuneus and calcarine and higher VMHC in the lentiform nucleus and caudate nucleus (LNCN) than those without CI. Furthermore, the VMHC in the LNCN was negatively associated with the global function and the memory function in WMH subjects.

**Conclusion:**

The enhanced VMHC in the LNCN was associated with the development of CI in individuals with WMH. This finding may contribute to the exploration of surrogate markers for the CI caused by WMH.

## Introduction

White matter hyperintensities (WMH) is commonly detected on magnetic resonance imaging (MRI) scans of the brain in the elderly. As the population ages, as much as 72–96% of the elderly would show WMH (Zhuang et al., 2018; Lampe et al., 2019). WMH in the elderly usually reflects axonal loss and demyelination resulting from chronic ischemia related to cerebral small vessel disease (Gouw et al., 2011). Many evidences have shown that the burden of WMH is negatively associated with cognitive function (Prins and Scheltens, 2015; Alber et al., 2019; Wu et al., 2019), and the increase of WMH burden is significantly paralleled by cognitive decline (LADIS Study Group, 2011; Schmidt et al., 2012). On the other hand, the cognitive performance of individuals with WMH tends to vary considerably, and a portion of these individuals even maintain normal cognitive function (Stern, 2002; Jokinen et al., 2016). The mechanisms underlying the high heterogeneity remain relatively unknown. Exploring the mechanisms is of value to the identification of surrogate markers related to the cognitive impairment (CI) in these individuals, thus directing the prevention of CI.

Functional magnetic resonance imaging (fMRI) technique detects alterations in synchronous activities of functionally related areas, also named as functional connectivity (FC), associated with aging or pathology. Voxel-mirrored homotopic connectivity (VMHC) refers to the voxel-based intrinsic FC between homotopic areas across hemispheres. VMHC reliably and reproducibly measures interhemispheric communication underlying the coherent cognitive function and behavior. A study on healthy individuals found that the VMHC in frontal, parietal, and temporal regions decreased with aging, and correlated with cognitive decline (Zhao et al., 2020). Other studies showed that the abnormal VMHC in frontal, parietal, or temporal regions was significantly associated with CI in individuals with stroke, Alzheimer’s Disease (AD), or type 2 diabetes mellitus (Li et al., 2018; Yao et al., 2020; Zhang et al., 2021). On the other hand, less evidence on the relationship between VMHC and cognitive function has been shown in individuals with WMH, despite that WMH may disrupt white matter tracts that connect hemispheres. Exploring the pattern of VMHC and its relationship with cognitive function in individuals with WMH may contribute to the identification of surrogate markers for CI.

The present study enrolled WMH subjects without CI, WMH subjects with CI, and healthy subjects (HS), and all subjects underwent multimodal MRI scanning and neuropsychological testing. We aimed to (1) identify the difference in the VMHC patterns among the three groups; (2) determine the relationship between the VMHC and cognitive function.

## Materials and Methods

### Subjects

The present study enrolled 138 subjects with WMH and 92 HS subjects. All the subjects were recruited from the Department of Neurology in The Affiliated Drum Tower Hospital of Nanjing University from January 2017 to December 2020. All the subjects have provided informed consent. The study had been approved by the Ethics Committee of The Affiliated Drum Tower Hospital of Nanjing University Medical School. All subjects underwent neuropsychological tests and multimodal MRI scans. As described previously (Chen et al., 2019), the inclusion criteria for subjects with WMH were: (1) age >50 years; and (2) presence of WMH on brain MRI (Fazekas grade 1∼3), no cerebral microbleeds or recent subcortical infarction. A HS group included those cognitively normal [measured by Montreal Cognitive Assessment (MoCA)] participants showing no clinical symptoms of cerebral small vessel disease, no presence of visible WMH on MRI (Fazekas grade 0), and no other MRI presentative characteristics of cerebral small vessel disease. The exclusion criteria were: (1) history of ischemic stroke with cerebral infarction diameter >15 mm, or cardiogenic cerebral infarction; (2) other cognitive disorders such as AD, Parkinson’s disease, Lewy body dementia, etc.; (3) intracranial hemorrhage, brain trauma, brain tumor and mental system disease, and severe somatic diseases, such as thyroid disease, anemia, malignant tumor, etc.; and (4) diseases such as multiple sclerosis, radiation brain injury, and other white matter diseases caused by poisoning, immunity, metabolism, infection and other factors.

### Neuropsychological Assessments

All the participants completed a series of neuropsychological tests. Neuropsychological assessments included global cognitive function, memory function, executive function, visuospatial ability and information processing speed. Global cognitive function was measured using the Mini-Mental State Examination (MMSE) and MoCA. An Auditory-verbal Learning Test-delayed recall (AVLT-DR) and a Rey-Osterrieth complex Figure Test (CFT) with its 20-min delayed recall (CFT-DR) were used to measured memory function. A Stroop Color and Word Test C (Stroop C) and Trail Making Tests (TMT)-B were used to measure executive function. An immediate recall of CFT was used to measure visuospatial performance. A Stroop Color and Word Test A (Stroop A) and TMT-A were used to measure information processing speed. Raw data of each neuropsychological test (except the MoCA and MMSE) were Z-transformed according to the following equation:


$$Z_{i}=\frac{\left(r_{i}-m\right)}{S}$$

*Z_i_* represents the *Z* scores for the *i*th subject, *r*_*i*_ represents the raw score for the *i*th subject, *m* represents the average score for each test for all subjects, and *S* represents the standard deviation of the test scores for all subjects. We performed Z-transformation across all subjects in all groups, and then the *Z*-transformed values of the relevant neuropsychological tests were averaged to obtain each cognitive domain. The purpose of the *Z*-transform is to facilitate the unification of different neuropsychological test data into a unified cognitive domain data. Due to the high sensitivity of the MoCA test for CI (Nasreddine et al., 2005), WMH subjects were diagnosed with CI when MoCA scores was ≤19 (education years: 1–6) or ≤24 (education years ≥7). According to the scores of neuropsychological tests, all subjects with WMH were divided into a WMH without CI group (*n* = 82) and a WMH with CI group (*n* = 56).

### Magnetic Resonance Imaging Procedures

As described previously (Ye et al., 2019), all subjects were scanned using a 3-Tesla magnetic resonance scanner (Ingenia 3.0T, Philips Medical Systems, Eindhoven, Netherlands) with a 32-channel head coil at the Drum Tower Hospital. All subjects were instructed to relax, close their eyes, and stay awake during scanning. Resting-state functional images, including 230 volumes, were acquired by a gradient-echo-planar imaging sequence: repetition time = 2,000 ms, echo time = 30 ms, flip angle = 90°, matrix = 64 × 64, voxel size = 3 mm× 3 mm× 3 mm, field of view = 192 mm× 192 mm, thickness = 4.0 mm, gap = 0 mm, and number of slices = 35.3D T1-weighted turbo fast echo sagittal images with high resolution were acquired with the following parameters: repetition time = 9.8 ms, echo time = 4.6 ms, flip angle = 8°, matrix = 256 × 256, field of view = 256 mm× 256 mm, thickness = 1.0 mm, gap = 0 mm, and number of slices = 192.3D fluid-attenuated inversion recovery (FLAIR) sagittal images were obtained with the following imaging parameters: repetition time = 4,500 ms, echo time = 344 ms, flip angle = 90°, matrix = 272 × 272, thickness = 1.0 mm, gap = 0 mm, and number of slices = 200.

### Magnetic Resonance Imaging Data Preprocessing and Static Voxel-Mirrored Homotopic Connectivity Analysis

The fMRI data were preprocessed using a toolbox for Data Processing Assistant for Resting-State fMRI (DPARSF) v2.3^1^ on Statistical Parametric Mapping software (SPM12)^2^. The preprocessing procedures included (1) removal of the first 10 time points to allow for T1 equilibration effects; (2) time correction for acquisition time delay among slices; (3) realignment to correct motion effects (subjects with head motion artifacts exceeding 2° in rotation or 2 mm in translation were excluded); (4) spatial normalization of the resulting images into the standard Montreal Neurological Institute (MNI) space and re-sample into a voxel size of 3 mm × 3 mm × 3 mm; (5) spatial smoothing with a Gaussian kernel of 6 mm× 6 mm× 6 mm; (6) nuisance covariates regression [white matter, cerebrospinal fluid, global signal, 6-head motion parameters, 6-head motion parameters at one time point earlier, and the 12 corresponding squared items (Friston 24-parameter model) as covariates]; and (7) linear detrending and temporal bandpass filter (0.01–0.1 Hz).

After preprocessing, the Pearson’s correlation coefficient between the residual time series of each voxel and its mirrored counterpart in the opposite hemisphere was calculated to obtain VMHC maps. Details of the VMHC calculations have been described in a previous study (Zuo et al., 2010). Detailed procedures are shown in Supplementary Material.

### White Matter Hyperintensities Segmentation and Quantification

The volume of WMH lesions was evaluated on T1 and T2-FLAIR images using the Lesion Segmentation Tool (LST) toolbox version 2.0.151^3^ for SPM12. Detailed procedures are shown in Supplementary Material. Notably, although the HS group included subjects with Fazekas grade 0 of WMH, the LST toolbox may detect tiny WMH lesions invisible on MRI. Thus, the HS group may have a small amount of WMH burden.

### Volume Assessment of Brain

As illustrated in a previous study (Ye et al., 2017), brain volume was estimated utilizing the Voxel-Based Morphometry 8 (VBM8) toolbox for SPM12. Detailed procedures are shown in Supplementary Material.

### Statistical Analysis

In the analysis for demographic characteristics, cognitive function, and volume data, normally distributed data were presented as mean ± standard deviation (SD) and analyzed using a one-way analysis of variance (ANOVA). Non-normally distributed data were presented as medians (quartiles) and analyzed using a Kruskal–Wallis test. The Chi-square test was applied in the analysis of gender. To improve the normal distribution of WMH volume data, the raw data were converted into log_10_ values.

In the analysis for VMHC data, an analysis of covariance (ANCOVA) was performed to identify the differences of VMHC maps among groups, controlling for age, gender, and education. The Resting State fMRI Data Analysis Toolkit (REST) 1.8 software^4^ was used in this procedure. The threshold was set at a corrected *P* < 0.01, determined by Monte Carlo simulation for multiple comparisons (voxel-wise *P* < 0.01). The full-width at half-maximum (FWHM) was estimated on VMHC maps and was used to calculate the threshold of the cluster size with the program AlphaSim in the REST software. Then, the average VMHC value of each region with significant group differences was derived in each subject. A post hoc test was conducted to reveal the detailed difference in the VMHC among the three groups.

We performed partial correlation analyses to test the relationship between the cognitive function and the VMHC values in each brain region with significant group differences, controlling for age, gender, and education. The Statistical Package for Social Sciences (SPSS) 19.0 software was used, and the significance was set at a *P* < 0.05.

## Results

### Demographic Characteristics

As described in Table 1, both the WMH without CI group and the WMH with CI group were significantly older than the HS group. No significant difference in gender was shown among the three groups. Although the WMH with CI group displayed significantly poorer performance in all cognitive domains than the WMH without CI group, there was no significant difference in gray matter atrophy rate, brain volume, or WMH volume between the two groups.

**TABLE 1:** Demographic, neuropsychological, and brain volume data.

Items	HS group (*n* = 92)	WMH without CI group (*n* = 82)	WMH with CI group (*n* = 56)	*F* or χ^2^	*P*-value
Age (year)	60.32 ± 7.42	64.63 ± 7.6	64.77 ± 8.3	8.891	**< 0.001^a^,^b^**
Male (%)	51 (55.43)	40 (48.78)	24 (42.86)	1.136	0.323
Education (year)	12 (9–16)	12 (9–15)	9 (9–12)	–	**0.026^b^**
MMSE	29 (28–30)	29 (28–30)	28 (26–29)	–	**< 0.001^b^,^c^**
MoCA	27 (25–28)	26 (25–27)	22 (19–23)	–	**< 0.001^b^,^c^**
Memory	0.38 ± 0.76	0.18 ± 0.65	−0.19 ± 0.70	11.261	**< 0.001^b^,^c^**
Executive function	0.34 (−0.20–1.00)	0.25 (−0.28–0.73)	−0.26 (−0.65–0.07)	–	**< 0.001^b^,^c^**
Visuospatial ability	0.56 (0.01–1.11)	0.29 (−0.26–0.77)	−0.26 (−0.53–0.29)	–	**< 0.001^b^,^c^**
Processing speed	0.42 ± 0.77	0.34 ± 0.76	−0.25 ± 0.7	15.001	**< 0.001^b^,^c^**
Gray matter atrophy (%)	41.35 ± 1.91	41.79 ± 2.16	41.02 ± 2.14	2.441	0.089
Brain volume (ml)	1350.4 ± 123.57	1344.88 ± 104.1	1340.15 ± 126.83	0.137	0.872
LogWMH	−0.03 ± 0.50	0.49 ± 0.31	0.54 ± 0.34	50.393	**< 0.001^a^,^b^**

*Education, MMSE, MoCA, executive function, and visuospatial ability data are presented as medians (quartiles) and were analyzed using a Kruskal–Wallis test. Chi-square test was applied in the comparisons of gender. Other data are presented as mean ± stand deviation (SD) and were analyzed using a one-way ANOVA. A log_10_ transformation was performed on the WMH volume data to improve the normal distribution of the data. Significance is highlighted in bold (P < 0.05).*

*^a^P < 0.05, differs between WMH without CI group and HS group.*

*^b^P < 0.05, differs between WMH with CI group and HS group.*

*^c^P < 0.05, differs between WMH without CI group and WMH with CI group.*

*CI, cognitive impairment; HS, healthy subjects; MMSE, Mini-Mental State Examination; MoCA, Montreal cognitive assessment; WMH, white matter hyperintensity.*

### Difference in Voxel-Mirrored Homotopic Connectivity Among Groups

**FIGURE 1:**
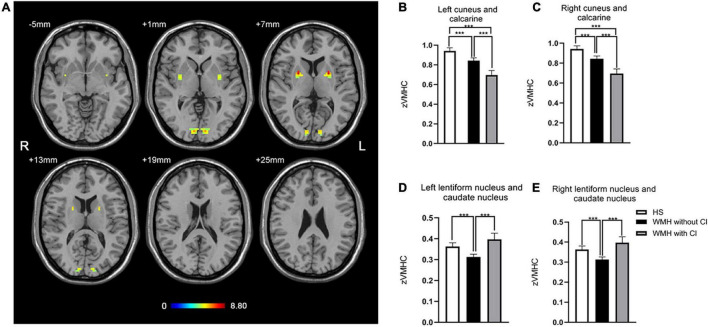
The differences in VMHC among groups. **(A)** Statistical maps showed VMHC differences in the bilateral cuneus and calcarine and the bilateral LNCN among the WMH without CI group, the WMH with CI group, and the HS group. The threshold was set at a corrected *P* < 0.01, determined by Monte Carlo simulation for multiple comparisons (voxel wise *P* < 0.01, cluster size >1,161 mm^3^). The color bar indicates the *F*-value. **(B,C)** In the bilateral cuneus and calcarine, both the WMH without CI group and the WMH with CI group had lower VMHC than the HS group, and the WMH with CI group had even lower VMHC than the WMH without CI group. **(D,E)** In the bilateral LNCN, the WMH without CI group displayed lower VMHC than the HS group, and the WMH with CI group had comparable VMHC to the HS group and higher VMHC than the WMH without CI group. Histogram indicated mean value and standard error of VMHC. ***P < 0.001. CI, cognitive impairment; HS, healthy subjects; LNCN, lentiform nucleus and caudate nucleus; VMHC, voxel-mirrored homotopic connectivity; WMH, white matter hyperintensity.

As shown in Figure 1A, the group difference of VMHC was shown in the bilateral cuneus and calcarine and the bilateral lentiform nucleus and caudate nucleus (LNCN) (*P* < 0.01, FWHM = 5.6 mm, cluster size >1,161 mm^3^, Monte Carlo corrected at the cluster-level). Specifically, in the bilateral cuneus and calcarine, both the WMH without CI group and the WMH with CI group had lower VMHC than the HS group, and the WMH with CI group had even lower VMHC than the WMH without CI group (Figures 1B,C). In the bilateral LNCN, however, only the WMH without CI group displayed lower VMHC than the HS group, and the WMH with CI group had comparable VMHC to the HS group and higher VMHC than the WMH without CI group (Figures 1D,E). Detailed coordinate information on the regions described above is available in Table 2.

**TABLE 2:** Brain regions with significant differences in VMHC between groups.

Brain regions	Peak MNI coordinates *x*, *y*, *z* (mm)	Peak *F*-value	BA	Cluster size (mm^3^)
Left cuneus and calcarine	−9, −90, 3	8.0726	17, 18	1161
Right cuneus and calcarine	9, −90, 3	8.0726	17, 18	1161
Left lentiform nucleus and caudate nucleus	−24, 3, 6	8.7673	–	891
Right lentiform nucleus and caudate nucleus	24, 3, 6	8.7673	–	891

*The thresholds were set at a corrected P < 0.01 determined by Monte Carlo simulation for multiple comparisons (voxel-wise P < 0.01). BA, Brodmann area; MNI, Montreal neurological institute.*

### The Association of Voxel-Mirrored Homotopic Connectivity With Cognitive Function

**FIGURE 2:**
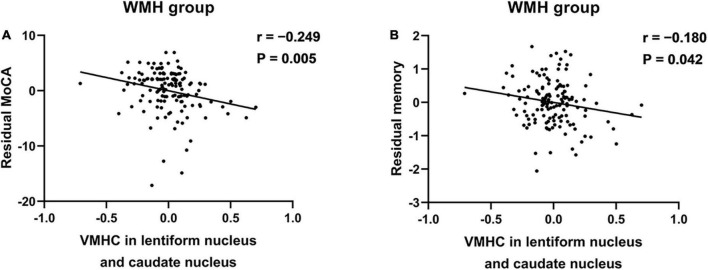
The association between the VMHC in the LNCN and cognitive function in WMH subjects. **(A)** The VMHC was negatively associated with the MoCA scores. **(B)** The VMHC was negatively associated with the memory function. The significance level for correlation analyses was set at *P* < 0.05. Partial correlation analyses were conducted between the residuals of variables after regression on the nuisance variables. LNCN, lentiform nucleus and caudate nucleus; MoCA, Montreal cognitive assessment; VMHC, voxel-mirrored homotopic connectivity; WMH, white matter hyperintensity.

In WMH subjects, the VMHC in the bilateral LNCN was significantly negatively correlated with both MOCA scores (*r* = −0.249, *P* = 0.005) and memory function (*r* = −0.180, *P* = 0.042) (Figures 2A,B).

## Discussion

The main findings of this study were as follows: (1) compared with WMH subjects without CI, those with CI displayed decreased VMHC in the cuneus and calcarine but increased VMHC in the LNCN; (2) the VMHC in the LNCN was associated with global function and memory function in WMH subjects. These findings contribute to the understanding of the relationship between interhemispheric connectivity and the cognitive heterogeneity in subjects with WMH.

Voxel-mirrored homotopic connectivity reflects the major role of interhemispheric communication in the integration of brain function underlying coherent behavior and cognition (Kelly et al., 2011). Interhemispheric coordination is of great importance for cognitive processes and handling complicated tasks (Belger and Banich, 1992; Wang et al., 2013). Ding et al. (2015) found that patients with subcortical vascular cognitive impairment displayed decreased VMHC in bilateral lingual gyrus, putamen, and precentral gyrus than patients with subcortical cerebrovascular disease and normal cognitive function. The decreased VMHC in the occipital regions was also shown in the present study, but we did not find altered VMHC in the putamen and precentral gyrus. This divergence may be due to the different grouping method and inclusion criteria for subjects. The striatum, including the caudate nucleus and the lentiform nucleus, is involved in motivation (Wolfram et al., 1997), implicit learning (Packard and Knowlton, 2002; Graybiel, 2008), inhibitory control, working memory, and set-shifting/flexibility (Grahn et al., 2008; McNab and Klingberg, 2008). The striatum is involved in integrating information from multiple cortical regions, thereby constituting a network that underlies decision making (Nagano-Saito et al., 2014). As shown in previous studies, the dysfunction of the striatum was associated with CI in multiple conditions or disorders (Asensio et al., 2010; Bailey and Goldman, 2017). Lewis et al. (2003) revealed that selective impairments in working memory and executive dysfunction were associated with reduced activity in the striatum in patients with Parkinson’s disease. Our findings support the role of the striatum in the development of CI in subjects with WMH.

In this study, the WMH without CI group displayed lower VMHC in the LNCN than both the HS group and the WMH with CI group, and no significant difference was shown between the latter two groups. This may suggest a U-shape curve of the VMHC in the LNCN related to the CI in subjects with WMH; that is, the VMHC may decrease significantly in WMH subjects with normal cognitive function, but then increase with the onset of CI. The decreased VMHC in WMH subjects with normal cognitive function may reflect the impaired inter-hemispheric connectivity related to disrupted white matter tracts. Then, with the onset of CI, compensatory enhancement in interhemispheric connectivity may be induced by the functional deterioration. The U-shaped curve of FC was also shown in previous studies. Meng et al. (2018) founded that compared with healthy control subjects, the baseline FC in the nucleus basalis of Meynert decreased in patients with mild cognitive impairment but increased in patients with AD and treated with cholinesterase inhibitor. Taya et al. (2018) explored global and local characteristic (clustering coefficient, normalized clustering coefficient, characteristic path length, normalized characteristic path length, and small-worldness) of an electroencephalogram-based network during performing a piloting task. They showed that the characteristic path length of the network firstly decreased and then increased during the training on a piloting task (Taya et al., 2018). A possible explanation for the U-shaped curve is that the connectivity between brain regions is disrupted by brain pathology or unfamiliar tasks, followed by enhanced connectivity to maintain cognitive performance after adapting to the pathology or tasks.

In WMH subjects, the VMHC in the LNCN was negatively correlated with both global function and memory function. The characteristic changes of the VMHC in the LNCN may be involved in the development of CI in subjects with WMH. On the other hand, comparable WMH volume, brain volume, and gray matter atrophy rate were shown between the two groups and could not be used to explain the difference in cognitive function. Thus, the altered VMHC may be more relevant to the CI and might contribute to the exploration for potential imaging biomarkers for the CI in individuals with WMH.

Both the WMH with CI group and the WMH without CI group showed decreased VMHC in the bilateral occipital lobes relative to the HS group, and the VMHC in the WMH with CI group was even lower than the WMH without CI group. The occipital cortex is mainly involved in visual information processing (Thiebaut de Schotten et al., 2014) and is also related to multiple functions such as memory (Cansino et al., 2017) and motor perception (Hidaka et al., 2017). The dysfunction of the occipital lobe affects not only the processing of visual information but also the performance of various cognitive tasks (Tohid et al., 2015). Zhang et al. (2021) found significantly decreased VMHC in the occipital lobe in patients with type 2 diabetes mellitus. And the decreased VMHC was associated with poor global function. Wang et al. (2015) showed that AD patients had significantly weaker VMHC in the occipital lobe than mild cognitive impairment subjects, and the VMHC in the occipital gyrus was positively correlated with the cognitive performance. Although we did not find significant association of the VMHC in the occipital lobe with cognitive function in subjects with WMH, the group difference in the VMHC between the two WMH groups might suggest a role of the VMHC in the development of CI.

Our study has several limitations. First, periventricular WMH burden and deep WMH burden were not analyzed in the present study, despite that periventricular WMH and deep WMH reflect different etiological and functional features (Kim et al., 2008). Although no significant difference in the total WMH burden was shown between the two WMH groups, there might be difference in periventricular WMH burden or/and deep WMH burden underlying the cognitive differences between groups. Second, in the present cross-sectional study, only the association relationships rather than the causal relationships were obtained between the VMHC alterations and cognitive function. Longitudinal studies would be helpful for exploring the causal relationships. Finally, the diagnosis of CI was based on clinical criteria, and we did not assess pathological markers to exclude other cognitive disorders. The CI might not be caused by the WMH burden in some cases but by other pathologies, e.g., AD pathology.

## Conclusion

The increased VMHC in the striatum was related to the presence of CI in subjects with WMH. The abnormal interhemispheric connectivity may be associated with the cognitive heterogeneity in individuals with WMH and may contribute to the exploration of surrogate markers for CI in these individuals.

## Data Availability Statement

The original contributions presented in this study are included in the article/Supplementary Material, further inquiries can be directed to the corresponding author/s.

## Ethics Statement

The studies involving human participants were reviewed and approved by the Ethics Committee of The Affiliated Drum Tower Hospital of Nanjing University Medical School. The patients/participants provided their written informed consent to participate in this study.

## Author Contributions

QY and YX contributed to the conceptualization, project administration, and writing and review. HZ contributed to the research project conception, data analysis, and writing – original draft. RQ contributed to the acquisition of the neuropsychological test. YC contributed to the acquisition of the data and statistical analysis. LH contributed to the acquisition of the data. PS and HX contributed to the acquisition of the image data. All authors approved the final version of the manuscript.

## Conflict of Interest

The authors declare that the research was conducted in the absence of any commercial or financial relationships that could be construed as a potential conflict of interest.

## Publisher’s Note

All claims expressed in this article are solely those of the authors and do not necessarily represent those of their affiliated organizations, or those of the publisher, the editors and the reviewers. Any product that may be evaluated in this article, or claim that may be made by its manufacturer, is not guaranteed or endorsed by the publisher.

## Footnotes

1
http://resting-fmri.sourceforge.net

2
http://www.fil.ion.ucl.ac.uk/spm

3
www.statistical-modelling.de/lst.html

4
http://restfmri.net/forum/index.php
