# Supplementary material for: The Enhanced Interhemispheric Functional Connectivity in the Striatum Is Related to the Cognitive Impairment in Individuals With White Matter Hyperintensities
Source: Front Neurosci. 2022 Jun 28;16:899473. doi: 10.3389/fnins.2022.899473 (PMC9273905; doi:10.3389/fnins.2022.899473)
Supplement: Supplementary file 2 [file Data_Sheet_2.DOCX]

**Supplementary Information**

**1 Materials and methods**

**1.1 MRI data preprocessing and static VMHC analysis**

As described previously (Zuo et al., 2010), the preprocessing procedures included: 1) a mean normalized T1 image was created by averaging normalized T1 images and was then averaged with its left–right mirrored version to create a group-specific symmetrical template. 2) the nonlinear registration to standard space for every subject was refined using the symmetrical template, and the refined transformation to the symmetrical brain template was applied to every subject's residual functional data. 3) the Pearson’s correlation coefficient between the residual time series of each voxel and that of its symmetrical interhemispheric counterpart was calculated and Fisher Z-transformed. To control the potential impacts of white matter, cerebrospinal fluid and other non-grey matter locations on homotopic functional connectivity, Fisher Z-values were weighted by the voxel-wise grey matter tissue probability. The resultant values were referred to as the VMHC and were used for subsequent group-level analyses.

**1.2 WMH segmentation and quantification**

The T1-weighted images were segmented into gray matter, white matter and cerebrospinal fluid using the lesion growth algorithm (Schmidt et al., 2012), and this information was combined with corresponding T2-FLAIR intensities to compute lesion belief maps. Three experienced radiologists determined a pre-chosen initial threshold (κ = 0.15) to these maps. Then the initial binary lesion maps were obtained, and were subsequently grown along voxels that appear hyperintensity on the T2-FLAIR images. The individual WMH volume was presented in an HTML report.

**1.3 Volume assessment of brain**

First, the T1 images were segmented into grey matter, white matter and cerebrospinal fluid. Second, the segmented images were normalized to the MNI template using a non-linear and affine spatial normalization and re-sampled to a voxel size of 1.5 × 1.5 × 1.5 mm. Third, Jacobian modulation was applied to the segmented images, which could be incorporated to compensate for the effect of spatial normalization. Fourth, the extracted grey matter, white matter and cerebrospinal fluid sets were smoothed with an 8-mm full width at half maximum Gaussian filter to decrease the effects of individual variation in gyral anatomy and to increase the signal-to-noise ratio. Finally, grey matter volume, white matter volume and cerebrospinal fluid volume were obtained in each subject. Grey matter atrophy is a calculation of grey matter volumes divided by the brain volume.

**References**

Schmidt, P., Gaser, C., Arsic, M., Buck, D., Förschler, A., Berthele, A., et al. (2012). An automated tool for detection of FLAIR-hyperintense white-matter lesions in Multiple Sclerosis. *Neuroimage* 59, 3774–3783. doi:10.1016/j.neuroimage.2011.11.032.

Zuo, X.-N., Kelly, C., Di Martino, A., Mennes, M., Margulies, D. S., Bangaru, S., et al. (2010). Growing together and growing apart: regional and sex differences in the lifespan developmental trajectories of functional homotopy. *J Neurosci* 30, 15034–43. doi:10.1523/JNEUROSCI.2612-10.2010.
